# First year nursing students’ reflections about developing their verbal nursing skills during their nursing education in China: a qualitative study

**DOI:** 10.3389/fpubh.2023.1149512

**Published:** 2023-05-05

**Authors:** Xiaoling Zhu, Mikael Rask, Hongbo Xu

**Affiliations:** ^1^Wenzhou Medical University, School of Nursing, Wenzhou, Zhejiang, China; ^2^Linnaeus University, School of Health and Caring Sciences, Växjö, Sweden

**Keywords:** verbal nursing skills, nurse–patient interaction, nursing education, content analysis, qualitative study

## Abstract

**Objective:**

The aim of the study is to explore the expectations of a group of first-year nursing students in China about developing their verbal and social interactional skills during their nursing education.

**Background:**

Nursing students’ communication skills were not fully developed in China. Students have many challenges to face regarding developing their nursing skills, especially interaction skills, when they start their education.

**Design:**

A qualitative design was used in this research.

**Method:**

Twelve second-semester undergraduate nursing students were interviewed on the basis of purposive sampling, and qualitative content analysis was employed.

**Results:**

The main theme was ‘facilitating a caring nurse–patient relationship’ and ‘using a knowledge base for performing nursing care’. The first theme comprises two sub-themes, ‘caring approach’ and ‘helping and involving the patient in care’, with three and two categories, respectively. The second theme comprises the two sub-themes, ‘knowledge needed to be able to understand the patient’ and ‘health and treatment information’, with three and two categories, respectively.

**Conclusion:**

A synthesis of both knowledge and practice is needed to improve the nursing students’ interaction and professional skills during their nursing education.

## 1. Introduction

With the view of the human as a biopsychosocial and spiritual being, nursing practice is increasingly emphasizing values and human dignity. As caring being the core of nursing, the ability to provide patient-centered and value-based care is an essential quality for nurses (1). And cultivating nurses with humanistic care attributes has also become an important goal of nursing education. A review (2) summarized that humanistic care is a multifaceted process during which the nurses provide a healing environment for the patient to ensure the patient’s human dignity and prioritize it. The nurse who excelled in clinical literacy tries to help the patients move toward positive attitude and independence. And this process could be realized through establishing constructive and dynamic interaction.

Therefore, developing interactional skills in nursing is considered to be a key element for a caring relationship between nurses and patients (3, 4), and the development of these skills during their nursing education is essential for nursing students for their encounters with their future patients (5). It is often stated as being difficult to learn just by reading textbooks, but instead requires a variety of different types of practical training (6, 7). Bach and Grant (3) maintain that adequate and effective interactions for establishing a caring relationship are considered as the first steps towards the nursing profession in nursing education. However, the students’ abilities in this particular area are poorly developed in the Chinese nursing education system (8, 9).

People learn things in relation to their experiences (10). Attention has to be paid to the students’ thoughts and feelings, and to supporting the student’s knowledge construction in order to create a supportive learning context. The capability of the Chinese nursing education to incorporate the students’ expectations for developing their interaction skills may affect the degree to which the education is accepted and the effectiveness of the students’ learning (11). A lack of understanding of the students’ experiences can lead to a mismatch of information between faculty and students, and a failure to achieve the results expected by faculty. This may lead to a psychological fallout and a change of major during the students’ nursing education.

## 2. Background

Nurses are expected to be able to manage several difficult situations, including giving medical treatment and interacting with patients. It has been shown in several studies in European and Asian countries that nurses’ interactional skills are perceived as important for giving successful treatment (12–15). Aspects that patients perceive as important for the quality of care include being listened to and respected and meeting nurses who show a caring and understanding attitude (16–18). From an existentialist point of view, the importance of nurse–patient interaction and a good caring relationship reflects people’s pursuit of human value and dignity.

A good interactional skill is helpful for learning in the clinic and needs to be trained during nursing education. Nursing students who possess this skill find themselves more confident and perform better in their learning and caring for the patients when they are in the clinic (19). However, many students in China face a major challenge in establishing a good, caring relationship, due to an inherent self-centered and inconsiderate nature based on excessive parental affection as the only child in their family. Providing them with training that focuses on how to interact with other people with empathy based on mutuality is a necessary element in the nursing education (20).

Many requisites are required for the establishment and maintenance of the nurse–patient relationship according to nursing theories. Firstly, the view of human beings is intertwined with interactions in a caring process, in which the two sides exchange their expectations and beliefs on the basis of mutual trust (21). The nurse–patient relationship is also founded on the nurse’s concern for the patient’s well-being, enabling the patient to alleviate suffering, recreate balance and find meaning in life in a state of illness (1). Moreover, it is important that the nurse balance her professional power over the patient in this asymmetric relationship (22) and make the relationship develop from a superficial to a deeper level (1, 23).

Nursing educators in China have started to focus on the development of students’ interactional, emotional, ethical and other humanistic care abilities, but more effort still need to be invested. Nursing teachers in China pay less attention to nurse–patient communication than to the basic theory, knowledge and skills of nursing due to the influence of the traditional medical teaching mode (19, 24). Studies showed that humanities courses accounted for less than 9% of the total teaching hours in nursing colleges in China, and the curriculum differed between schools and lacked a systematic and scientific basis (24–26). Educational methods are mainly traditional didactic and experimental imitation, with some schools adding narrative teaching, role-playing and case discussions in recent years, but generally lacking integration with social practice and attention to the needs of students’ individual development (25–27).

Nursing students, therefore, are facing significant limitations in developing a humanistic approach (19). Chen and Wang (8) identified a number of problems in the communication between nursing students and patients: weak communication awareness, a lack of communication skills, an inability to integrate theory with practice, the majority of students having difficulties adapting to the caring environment and a lack of self-confidence in communication with the patients.

Approaches such as clinical learning, reflection and health care experiences have been found to influence students’ ability to interact with patients and contribute to harmonious and trusting relationships. For example, clinical practice can help students understand how their theoretical knowledge can be used in their future daily work as nurses; a reflective approach during the clinical studies increases students’ flexibility to interact with patients (6, 7). Furthermore, the graduate study experiences, previous hospital experience and nursing experience may lead to greater compassion among the nursing students towards the suffering of the patients and strengthen their self-confidence as a healthcare provider (21, 28).

Differences were found in the first-year nursing students’ perceptions of their verbal and social interaction skills across cultures and educational systems in our previous study (21). We carried out the present study from a phenomenological perspective in order to gain a greater understanding of this phenomenon among Chinese first-year students and present another picture of its meaning. We hope to contribute valuable knowledge through the lived experiences of students in this qualitative study in comparison to the result from our previous quantitative study.

### 2.1. Aim

The aim of the study is to explore the expectations of a group of first-year nursing students in China about developing their verbal and social interactional skills during their nursing education.

## 3. Materials and methods

### 3.1. Design

For any student’s expectation of her/his interactional skills, regardless of individual differences, there are essential structures that make it up. It is known that qualitative approach are feasible for settings in which the experience of individuals is of concern and can provide evidence for developing applicable knowledge and affecting policy making (29). This study used a qualitative, descriptive methodology, based on an inductive content analysis of interviews, to find a common structure of the experiences of the first-year nursing students at a medical university in southeast of P.R. China. To reach the objectivity and be faithful to what it is, we strived for descriptive identification of the phenomenon understudy, eliminating the researcher’s personal inclinations and predispositions. Thirty-two items of Tong qualitative research report were observed (COREQ) (30).

### 3.2. Ethical considerations

The performance of this study was guided by the Standards and Operational Guidance for Ethics Review of Health-related Research with Human Participants (31) and approved by the institutional review board of the Wenzhou Medical University (No. 2022-019). Ethical approval was not required in China at the time the study was conducted, so it was obtained after the data collection was completed. Written informed consents were signed by the participants prior to the data collection. They were informed about the aim and design of this study, and all of them consented to participate in the study on a voluntary basis. They were also informed that they had the right to withdraw at any time without clarifying the reasons and that their withdrawal would not have any negative impact on them. Confidentiality was assured; no names or places are mentioned in the results.

### 3.3. Setting and sample

Twelve Chinese undergraduate nursing students were selected in a process of purposive sampling. The participants’ demographic information is shown in Table 1. They were numbered A1–A12. Their average age was 20.42 ± 0.64. Since the total number of male nursing students (*n* = 52) in semester two was much lower than the number of female students (*n* = 285), we chose less male participants than the female participants. And most of them were from urban areas and had no experience related with nursing. Four of them (A7, A9, A10, and A12) had some experience interacting with nurses as outpatients or as relatives of patients. They were in the second semester of their four-year nursing education and had completed the courses of nursing psychology and introduction to nursing in their first semester prior to the interview. They had learned some nursing theory and knowledge about interpersonal relationships, and they had been to the hospital for an internship, where they practicing their knowledge from the two aforementioned courses on two occasions. Furthermore, they had also studied the humanities a little after being admitted to the university.

**Table 1:** Basic information of participants (*n* = 12).

Variable	Group	Frequency (participant)
Age	19	1 (A10)
	20	5 (A4, A5-A7, and A11)
	21	6 (A1-3, A5, A9, and A12)
Gender	Female	9 (A1-A3,A5-A6, and A9–A12)
	Male	3 (A4, A7, and A8)
Origin	Rural	5 (A5–A7, A9–A10)
	Urban	7 (A1–A4, A8, and A11–A12)
Experience related with nursing	Yes	4 (A7, A9–A10, and A12)
	No	8 (A1–A6, A8, and A11)

### 3.4. Data collection

All the 12 face-to-face interviews, without any dropout, were conducted by the first author who had known the participants well since they entered university. No changes were made to the interview guide after the first interview and its data were included in the analysis. The data was collected from March 12th to June 6th 2018 until no new information was discovered. Prior to each interview, the researcher made an appointment to confirm the interviewee’s choice of location and an uninterrupted, untimed interview. All the students talked Mandarin fluently. Each interview lasted from 30 to 72 min. A semi-structured interview guide was used, focusing on the student’s perception of verbal and social interaction with patients and the nurse–patient relationship. Examples of the questions: *Can you tell me about your thoughts about caring for patients? Can you give me an example or examples of these; How do you think relationships with patients are created? Can you give me an example or examples of these; What do you think it is like to talk to the patients about their feelings and thoughts? Can you give me an example or examples of these; Do you think it is difficult for you to do it?* Probing questions, such as ‘*Can you tell me more about that?’* and *‘Can you explain more?’,* were asked to gain in-depth data. Every interview was audio-recorded.

### 3.5. Data analysis

The audio-recorded interviews were transcribed verbatim and analyzed by performing a qualitative content analysis (32, 33). The text was read several times to gain a sense of the whole and become familiar with it. Meaningful units with relevance to the study were identified, condensed and labeled with codes (32). The analysis was performed by each author for comparison. The process continued, and various categorizations were tested several times. Different interpretations of the text were discussed among the researchers until consensus was reached. We also questioned and reflected on our preunderstandings during the analysis process, where the latent meaning of the text was also in focus. After member checking with two interviewees, an overarching theme with two sub-themes was confirmed based on the subcategories, categories and the text as a whole. Examples of the analysis process is presented in Table 2.

**Table 2:** Examples of content analysis process.

Meaning units	Condensed meaning units	Codes	Categories
I feel that if I communicate with a patient now, because I have not learnt that much, I can only let the patient know that I am genuinely taking care of him and sincerely want him to be healthy. I can only do my best to make him trust me. (A12)	To let the patient know that the nurse sincerely wants him to be healthy and to gain his trust	Be sincere	Trustful relationship
By mastering communication skills, you can subtly integrate the assessment into the daily conversation with the patient, and you can complete the work easily and effectively. (A2)	Integrate the assessment into the daily conversation	Be flexible in communication	Communication method

## 4. Results

Two main themes, 4 sub-themes and 10 categories were identified from the analysis (Table 3). The nursing students described what they believe is essential for developing verbal and social interaction skills during their nursing education to become skilled nurses.

**Table 3:** Presentation of theme, sub-themes, categories and sub-categories.

Themes	Sub-themes	Categories
Facilitating a caring nurse–patient relationship	Caring approach	Friendly and understanding approach
		Show interest
		Trustful relationship
	Helping and involving the patient in care	Helping the patient
		Inviting the patient
Using a knowledge base for performing nursing care	Knowledge needed to be able to understand the patient	Observe the patient
		Learn about the patient
		Communication method
	Health and treatment information	Inform the patient
		Teaching

### 4.1. Facilitating a caring nurse–patient relationship

This theme can be described as actions to facilitate a caring nurse–patient relationship by developing a caring approach combined with helping and involving the patient in her/his care. The caring approach can be understood as the skills that nurses need for their development as nurses, which includes showing the patients that you are prepared to interact with them by having a friendly and understanding approach and by showing interest in who the patient is or in her/his problems. The students describe the caring approach as being patient with the patient, expressing oneself as forthrightly as possible, sometimes using humor and being easy-going while trying to think from the patient’s position or perspective. They describe showing interest as listening attentively with an encouraging attitude while accompanying the patient and trying to have as good eye contact as possible in a suitable way. They also describe that it is important to try to develop a trusting relationship with the patients, which could be done by paying respect to the patient and being sincere.

*‘…when I meet a new patient, I must first let her/him know that I can empathise with her/his illness and I can understand him/her. If I cannot do that, I will find something we have in common. When we have this, then we can build a relationship through empathy’.* (A12)

*‘…you have to show as much kindness and friendliness as you can’.* (A10)

Helping and involving patient in her/his care is important for developing students’ skills in helping the patient and inviting the patient to take part in their care. The help could be in the form of trying to relieve physical discomfort, assisting the patient with solving economic problems, and building social relationships. The students indicate several aspects that they see as necessary to be able to stimulate the patients to take an active part in their care. The students state that, as a nurse, you have to take responsibility for initiating communication with the patient. Furthermore, they state that patients’ involvement in their care could be promoted by involving the patients in their disease management by communicating the patient’s condition, treatment plan and treatment progress in detail.

*‘When you go to check their physical condition, talk about their recent situation and how they feel. The patient will then feel that someone cares and that there is emotional resonance’.* (A3)

*‘Nurses are supposed to tell the patients how to take the medicine and how many bottles of saline to have in an intravenous drip. Just let them know these things. Tell them in the morning, and then they can schedule the day’.* (A1)

### 4.2. Using a knowledge base for performing nursing care

This theme can be described as the knowledge that nurses need to be able to understand the patient and provide information about health and treatment. It is more closely connected to the knowledge base that nurses need to have to be able to provide care. The nursing students discussed the importance of developing skills to obtain the knowledge needed for understanding the patient by communicating with and observing the patient in different ways. This could be done by focusing on the patient’s behavior and incorporating assessments into their daily conversations with the patients. They also talk about the need to improve their skills in learning about the patients’ physiology, psychological needs, living habits, social background and social contacts.

*‘By mastering communication skills, you can subtly integrate the assessment into the daily conversation with the patient, and you can complete the work easily and effectively’.* (A2)

*‘Talking about his daily life, beating around the bush to care about his physical condition, and then learning about his psychological condition by watching how he behaves in the conversation. At first, ask him a question, and then, as he speaks, observe his behaviour, for example, his words, eyes and movements’.* (A6)

They also express that they need to develop their skills in using different communication methods and learn about the patient by asking her/him, asking the family members and the doctor. Developing their skills in using open-ended and fixed questions and using questionnaires to support their communication are examples of these methods. The students describe that communication is essential for the patients’ recovery and should be gradual, frequent and in an easy-to-understand language. They also maintain that it is important to try to grasp the right time to communicate. Furthermore, they also describe it as being flexible in communication, communicating in accordance with the patient’s characteristics and condition.

*‘When talking with the patient about feelings and thoughts in the process of treatment, I will learn about his daily life first and then ask him about his intuitive feelings about the treatment gradually. Next, I would ask him about his thoughts on whether it is valid or not according to his intuitive feelings. Then, I would ask him about his thoughts on treatment and improve the treatment plan based on these, as well as some test results’.* (A5)

Furthermore, they must develop communication skills to teach and inform patients about different health and treatment issues. They spoke of education being delivered in sections, that they can use a case methodology and that they have to be flexible in their communication. Understanding the patient’s characteristics and condition is the most important and basic communication premise. Therefore, they must communicate drug effects and provide information about how medical administration is carried out, as well as the importance of exercise.

*‘Some aspects, such as exercise, diet and sleep, should not be discussed all at once, so I do not think I am going to mention them all at once. Maybe I will talk about the recent diet with him when he comes to ask me in the morning and then tell him about the methods of exercising when he takes an injection’.* (A10)

## 5. Discussion

To work as a nurse means, to a great extent, to interact with patients in various ways with the main aim to support patients’ health and well-being. However, studies have found that the new nurses lack the knowledge to understand the existential qualities that are both common and unique to different conditions and different patients (9, 23). It is therefore critical for the educational institutions to develop the communicating ability of the nurses and nursing students to help them provide quality care in the future.

The nursing students in our study confirm that both the facilitation of the caring relationship and the application of knowledge are required in their future work. It could be understood that they knew the importance of intertwining caring interactional skills with knowledge and are eager to learn how to facilitate caring relationships and the related knowledge of caring. Several studies, including our previous research, reported that the Chinese nursing students had a lower level of caring competence than seen among their foreign counterparts (34, 35). Essentially, this is a problem related to how the transfer of medical knowledge and humanistic care knowledge are balanced during nursing education. For a long period, China’s nursing education system has focused on the treatment of disease more than the development of humanistic competence (2). However, the theoretical underpinnings of nursing is holism and humanism. Nurse educators are required to make the students understand deeply that well-being is not merely the absence of disease, but a harmonious state on the basis of the interconnection among mind, body, spirit, and environmental and social conditions. And the nurse’s communication and deep engagement with a patient is the way in which the patient’s well-being could be provided.

The students regarded consideration, friendliness and understanding as equally important as showing interest in and building a trusting relationship with the patients. They knew that to give special attention to, cherish and appreciate the patients would allow love and caring to come together in a new form of deep transpersonal caring. It could be seen that they mainly gained this understanding from some of their life experiences, while a deep understanding of related knowledge or nursing theory on how to facilitate a caring relationship might be more important for them. The nursing humanistic theories are abstract and difficult for novice nursing students to understand, and applying these theories into practice is even harder (36). China has recently begun to promote ideological and political educations that emphasize morality and ethics, humanity and love (37). In this context, it is necessary to infuse the essence of caring theory into educational curricula and clinical practice models to develop a sense of intentional caring in students throughout their education (38).

The students described that helping a patient was not only about actively addressing her/his illness but also included many psychological and social problems and allowing the patient to take charge of her/his own treatment and arrangements. This somehow reflects their understanding that the human being is a whole person and that well-being encompasses existential dimensions of freedom and vulnerability (23). Nursing students thus need to learn how to provide value-based care before they encounter the patient’s lifeworld, to be able to promote patients’ physical, spiritual and existential well-being (21). The instructors are supposed to provide as much contextual information about the patient as possible in the classroom setting and challenge the students to provide care strategies in relation to the patient’s lifeworld.

Being able to gain knowledge about understanding the patients through verbal interaction and behavior observation and learning the communication methods and techniques are considered to be important aspects for the development of their caring capability, according to the students. Nurse educators have to make the students understand during their nursing education that the perception of every patient is unique as they have different values, culture, race, beliefs, past experiences and expectations (39). Each patient acts based on her/his perception of illness, well-being and other situations. Since the patient’s perception could be found through talking and observing behavior, a nurse is expected to ask the patient about the feelings and observe her/him in order to understand her/him better (40). A nurse should create a supportive environment for this process (41). This is the only way the nurse can understand the patients from the inside out and provide them with the help they need. The students admitted that though they remembered what they had learned about communication in the class, they currently lacked the ability to have more profound conversations with the patients, which could be achieved through ‘reflection in action’ and developing the knowledge that goes beyond the theoretical studies (8).

The students stated that the level of mastery of communication skills and professional knowledge in their interactions with the patients was what may affect the quality of care they provide to patients. Patients are the masters of their own health and they have the right to be informed about the knowledge of their disease and their own health conditions so that they can make decisions for themselves. The nurse works as a health educator, resource provider and counselor to work together with the patient to achieve her/his well-being (38). It is important that the meaning of well-being is not taken for granted, whether the nurse emphasizes the patient’s initiative or vulnerability (23). The students noted that more practice should be provided on the basis of their theoretical lessons so that they could develop interactive skills adapted to different contexts in real-situations. The use of scenario simulation and interactive panel discussions, the artificial intelligence-based virtual counseling application, and the intensive training of the tutoring system have proved helpful in improving students’ oral communication, psychological response and conflict resolution ability (42–44).

There are some limitations to this study. One is that the interviewer had been the interviewees’ teacher for 1 year, which may have made some of the interviewees a little nervous. A result could be that they might not express their ideas freely and fully during the interviews. The interviewer tried to have a convivial and relaxing conversation prior to reaching the relevant topic of this study to help them be relaxed. The other limitation is that the students had gained some knowledge about nursing psychology and communication after they entered university, so their original ideas about the nurse–patient interaction may have been affected. There is also a risk that some of the students could answer the questions in accordance with the textbooks they have read.

## 6. Conclusion

A concept that systematically reflects humanistic caring is lacked among the Chinese student compared to their foreign counterparts. They are eager to possess the qualities and abilities in the establishment of a good nurse–patient relationship, while gaining professional knowledge in their education. A harmonious caring relationship is supposed to be established through a considerate, understanding and friendly approach in helping the patients. They need to learn and understand the deeper mechanisms that enable this relationship and to apply them in the future.

## 7. Implications for nursing education

Nurse educators in China are suggested to systematically set the curriculum in accordance with caring theory and infuse its essence into the clinical practice models. Intentionally use a variety of teaching methods to challenge students’ ability to acquire background knowledge about patients and to provide nursing strategies that are relevant to patients’ lifeworld. Moreover, personalized teaching methods to cultivate the nursing students’ communication skills and professional abilities are also needed from a lifeworld perspective.

## Data availability statement

The raw data supporting the conclusions of this article will be made available by the authors, without undue reservation.

## Ethics statement

The studies involving human participants were reviewed and approved by Institutional Review Board of Wenzhou Medical University. The patients/participants provided their written informed consent to participate in this study.

## Author contributions

XZ, HX, and MR conceived the study. XZ collected data. XZ, HX, and MR conducted data analysis. All authors contributed to the article and approved the submitted version.

## Funding

This work was supported by the Wenzhou Association of Science and Technology (grant number 2019KXCX-KT18) and Basic Scientific Research Funds of Wenzhou Medical University (KYYW202024).

## Conflict of interest

The authors declare that the research was conducted in the absence of any commercial or financial relationships that could be construed as a potential conflict of interest.

## Publisher’s note

All claims expressed in this article are solely those of the authors and do not necessarily represent those of their affiliated organizations, or those of the publisher, the editors and the reviewers. Any product that may be evaluated in this article, or claim that may be made by its manufacturer, is not guaranteed or endorsed by the publisher.

## References

[1] Watson J. Caring in Nursing Classics: An Essential Resource. New York, NY: Springer Publishing Company (2012). 336 p.

[2] Taghinezhad F Mohammadi E Khademi M Kazemnejad A. Humanistic Care in Nursing: concept analysis using Rodgers' evolutionary approach. Iran J Nurs Midwife. (2022) 27:83–91. doi: 10.4103/ijnmr.ijnmr_156_21, PMID: 35419263 PMC8997180

[3] Bach S Grant A. Communication and Interpersonal Skills in Nursing. Thousand Oaks, California: SAGE Publications, Inc. Learning Matters (2018). 288 p.

[4] Riley JB. Communication in Nursing. St. Louis, Missouri: Elsevier (2019). 350 p.

[5] Bullington J Söderlund M Bos Sparén E Kneck Å Omérov P Cronqvist A. Communication skills in nursing: a phenomenologically-based communication training approach. Nurse Educ Pract. (2019) 39:136–41. doi: 10.1016/j.nepr.2019.08.011, PMID: 31487674

[6] Fröberg M Leanderson C Fläckman B Hedman-Lagerlöf E Björklund K Nilsson GH. Experiences of a student-run clinic in primary care: a mixed-method study with students, patients and supervisors. Scand J Prim Health. (2018) 36:36–46. doi: 10.1080/02813432.2018.1426143, PMID: 29368978 PMC5901439

[7] Albinsson G Elmqvist C Hörberg U. Nursing students’ and lecturers’ experience of learning at a university-based nursing student–run health clinic. Reflective Pract. (2019) 20:423–36. doi: 10.1080/14623943.2019.1638242

[8] Li HL Liu XC Cai Q. Research progress on the communication ability education of nurses and patients. Chin Commun Doc. (2018) 34:6–8.

[9] Chi MZ Jian DL Tan XM Liu HJ. A preliminary study on the cultivation of humanistic quality of medical students. J Mod Med Health. (2015) 11:3345–6.

[10] Ekebergh M. Lifeworld-based reflection and learning: a contribution to the reflective practice in nursing and nursing education. Reflective Pract. (2007) 8:331–43. doi: 10.1080/14623940701424835

[11] Lu CN. Thinking on the cultivation of nurse-patient communication ability of nursing students. J Minxi Vocat Tech Coll. (2017) 19:100–3.

[12] Larsson Gerdin A Hellzén O Rising-Holmström M. Nurses' experiences of encounters in home care: a phenomenological hermeneutic study. Int J Qual Stud Health Well-being. (2021) 16:1983950. doi: 10.1080/17482631.2021.1983950, PMID: 34633907 PMC8525981

[13] Kullberg A Sharp L Dahl O Brandberg Y Bergenmar M. Nurse perceptions of person-centered handovers in the oncological inpatient setting: a qualitative study. Int J Nurs Stud. (2018) 86:44–51. doi: 10.1016/j.ijnurstu.2018.06.001, PMID: 29960895

[14] Farzi S Taleghani F Yazdannik A Esfahani MS. Communication culture in cancer nursing care: an ethnographic study. Support Care Cancer. (2022) 30:615–23. doi: 10.1007/s00520-021-06388-2, PMID: 34357456

[15] Wittenberg E Reb A Kanter E. Communicating with patients and families around difficult topics in cancer care using the COMFORT communication curriculum. Semin Oncol Nurs. (2018) 34:264–73. doi: 10.1016/j.soncn.2018.06.007, PMID: 30100368 PMC6156926

[16] Collet R Major M Van Egmond M Van Der Leeden M Maccow R Eskes A. Experiences of interaction between people with cancer and their healthcare professionals: a systematic review and meta-synthesis of qualitative studies. Eur J Oncol Nurs. (2022) 60:102198. doi: 10.1016/j.ejon.2022.102198, PMID: 36087381

[17] Alikari V Gerogianni G Fradelos EC Kelesi M Kaba E Zyga S. Perceptions of caring behaviors among patients and nurses. Int J Environ Res Public Health. (2022) 20:396. doi: 10.3390/ijerph20010396, PMID: 36612719 PMC9819105

[18] Marcinowicz L Taranta E. Perspectives of older patients on the qualities which define a "good family nurse": a qualitative study. Nurs Open. (2020) 7:814–21. doi: 10.1002/nop2.456, PMID: 32257269 PMC7113527

[19] Ying L Zhang GH Xu Y. The relationship between interpersonal trust and nurse-patient communication among undergraduate interns: the mediating role of interpersonal self-efficacy. Theory Pract Educ. (2019) 39:30–2.

[20] Zhang J Jiang JX Ye M. Research progress in nurse-patient communication of nursing students. J Clin Pathol Res. (2017) 12:2732–8.

[21] Albinsson G Carlsson-Blomster M Lindqvist G. Data from: in search of a caring relationship—nursing students' notions of interactions in the nurse-patient relationship. Nurse Educ Pract. (2021) 50:103154. doi: 10.1016/j.nepr.2020.102954, PMID: 33360788

[22] Travelbee J. Interpersonal Aspects of Nursing. 2nd ed. Philadelphia, PA: F. A. Davis Company (1971). 242 p.

[23] Dahlberg K Todres L Galvin K. Lifeworld-led health care is more than patient-led care: an existential view of well-being. Med Health Care Phil. (2009) 12:265–71. doi: 10.1007/s11019-008-9174-7, PMID: 19101822

[24] Deng F Ouyang YW Wang Z Yao Q. Survey on the doctor-patient communication education of medical students. Chongqing Med J. (2015) 9:1241–3.

[25] Fan YY Sun HY Chang GM. Higher nursing education calls for the return of humanistic care—review on humanistic care nursing education. J Nurs Train. (2019) 34:1257–66. doi: 10.16821/j.cnki.hsjx.2019.14.003

[26] Liu Q Zou JM Pan Y Chen X Ye F Zhong CT. The construction of an integrated higher vocational nursing humanistic care curriculum system in the context of healthy China. Modern Voc Educ. (2022) 7:46–8.

[27] Han J Li P He Y Wang B Yao M. Research on the teaching practice and effect of professional image course for nurses on improving nursing students' humanistic care ability. Health Voc Educ. (2023) 41:58–60. doi: 10.20037/j.issn.1671-1246.2023.01.19

[28] Belcher M Jones LK. Graduate nurses’ experiences of developing trust in the nurse–patient relationship. Contemp Nurse. (2019) 31:142–52. doi: 10.5172/conu.673.31.2.142 19379116

[29] Fitzpatrick R Boulton M. Qualitative methods for assessing health care. Qual Health Care. (1994) 3:107–13. doi: 10.1136/qshc.3.2.107, PMID: 10137583 PMC1055206

[30] Tong A Sainsbury P Craig J. Consolidated criteria for reporting qualitative research (coreq): a 32-item checklist for interviews and focus groups. Int J Qual Health Care. (2007) 19:349–57. doi: 10.1093/intqhc/mzm04, PMID: 17872937

[31] WHO. Standards and Operational Guidance for Ethics Review of Health-Related Research With Human Participants. Geneva, Switzerland: World Health Organization (2011). 41 p. 26269877

[32] Graneheim UH Lundman B. Qualitative content analysis in nursing research: concepts, procedures and measures to achieve trustworthiness. Nurse Educ Today. (2004) 24:105–12. doi: 10.1016/j.nedt.2003.10.001, PMID: 14769454

[33] Graneheim UH Lindgren BM Lundman B. Methodological challenges in qualitative content analysis: a discussion paper. Nurse Educ Today. (2017) 56:29–34. doi: 10.1016/j.nedt.2017.06.002, PMID: 28651100

[34] Lu MX Wang M Liu YQ Liu TT. Investigation on current situation and influencing factors of humanistic care ability of nursing undergraduates. China High Med Educ. (2020) 3:28–9.

[35] Lindqvist G Ge L Borg C Zhu XL Xu HB Safipour J. Nursing students’ perceptions of their verbal and social interaction skills in Sweden and China during their first semester. QANE-AFI. (2022) 8:1–20. doi: 10.17483/2368-6669.1308

[36] Luo JF Liao XL Wang D. Research on the theoretical basis and development trend of modern nursing in China. J PLA Nurs. (2015) 21:51–3.

[37] Qiao WB Zhu BB Yu ZL Wang W. Design and practice of health promotion and health education"curriculum ideological and political educations". Nurs Rehab. (2022) 10:79–81.

[38] Parker ME Smith MC. Nursing Theories and Nursing Practice. 5th ed. Pennsylvania, USA: F.A. Davis Co (2019). 576 p.

[39] Thomas A William S Andrea NP Eileen G. Peplau’s theory of interpersonal relations. Nurs Sci Q. (2017) 30:160–7. doi: 10.1177/0894318417693286, PMID: 28899257 PMC5831243

[40] McCance TV. Caring in nursing practice: the development of a conceptual framework. Res Theor Nurs Pract. (2003) 17:1541–6577. doi: 10.1891/rtnp.17.2.101.53174 12880216

[41] Covington H. Caring presence: providing a safe space for patients. Holist Nurs Pract. (2005) 19:169–72. doi: 10.1097/00004650-200507000-00008 16006831

[42] He BH Wang RJ Liu J. Application of the scene simulation and interactive subject-seminar teaching method in the training of the communication ability of nurse-patient relationship of higher vocational nursing students. J Bengbu Med Coll. (2017) 4:525–8. doi: 10.13898/j.cnki.issn.1000-2200.2017.04.032

[43] Shorey S Ang E Ng ED Yap J Lau LST Chui CK. Communication skills training using virtual reality: a descriptive qualitative study. Nurse Educ Today. (2020) 94:104592. doi: 10.1016/j.nedt.2020.104592, PMID: 32942248

[44] He ZF Feng YQ Yang JJ Ren HY Chen XX. Effectiveness evaluation of pre-job intensive training on the communication ability of nursing students. Educ Teach Forum. (2017) 21:48–9.

